# The RNA Content of Fungal Extracellular Vesicles: At the “Cutting-Edge” of Pathophysiology Regulation

**DOI:** 10.3390/cells11142184

**Published:** 2022-07-13

**Authors:** Tamires A. Bitencourt, André M. Pessoni, Bianca T. M. Oliveira, Lysangela R. Alves, Fausto Almeida

**Affiliations:** 1 Department of Biochemistry and Immunology, Ribeirao Preto Medical School, University of São Paulo, Ribeirao Preto 14049-900, SP, Brazil; tabitencourt@gmail.com (T.A.B.); andrepessoni1@gmail.com (A.M.P.); bianca_texeira@hotmail.com (B.T.M.O.); 2 Gene Expression Regulation Laboratory, Carlos Chagas Institute, Fiocruz, Curitiba 81350-010, PR, Brazil; lysangela.alves@fiocruz.br

**Keywords:** fungal infections, RNAs, communication, signaling, extracellular vesicles

## Abstract

The role of extracellular vesicles (EVs) in interkingdom communication is widely accepted, and their role in intraspecies communication has been strengthened by recent research. Based on the regulation promoted by EV-associated molecules, the interactions between host and pathogens can reveal different pathways that ultimately affect infection outcomes. As a great part of the regulation is ascribable to RNA contained in EVs, many studies have focused on profiling RNAs in fungal and host EVs, tracking their accumulation during infection, and identifying potential target genes. Herein, we overview the main classes of RNA contained in fungal EVs and the biological processes regulated by these molecules, portraying a state-of-the-art picture of RNAs loaded in fungal EVs, while also raising several questions to drive future investigations. Our compiled data show unambiguously that EVs act as key elements in signaling pathways, and play a crucial role in pathosystems. A complete understanding of the processes that govern RNA content loading and trafficking, and its effect on recipient cells, will lead to improved technologies to ward off infectious agents that threaten human health.

## 1. Introduction

Extracellular vesicles (EVs) are cell-derived structures delimited by lipid bilayers unable to auto-replicate. These are produced by organisms from virtually every kingdom, reverberating their conserved origin. These structures transport different cargoes, including polysaccharides, toxins, lipids, proteins, pigments, and nucleic acids [1,2].

According to their origin and size, EVs comprise different entities, such as apoptotic bodies, exosomes, and microvesicles [3,4]. Exosomes display sizes ranging from 40 to 100 nm in diameter, and have an endosomal origin, which is the consequence of plasmatic membrane fusion with multivesicular bodies (MVBs) [5]. Microvesicles and apoptotic bodies are produced by membrane budding, with sizes ranging from 50 nm to 1 µm and 50 nm to 5 µM, respectively [5,6]. Although many aspects of EVs’ biogenesis are not well understood, efforts to unveil their origins are in progress, hinting that these may be found by tracking proteins such as Ras-related protein GTPase Rab, sytenin-1, TSG101 (tumor susceptibility gene 101), ALIX (apoptosis-linked gene 2-interacting protein X), syndecan-1, ESCRT (endosomal sorting complexes required for transport) proteins, phospholipids, tetraspanins, ceramides, sphingomyelinases, and SNARE (soluble N-ethylmaleimide-sensitive factor (NSF) attachment protein receptor) complex proteins [6].

The first identification of fungal EVs was reported in 1973, through freeze-etching of *Cryptococcus neoformans* [7]. However, it took more than three decades to characterize the EV content in this species [8]. This work initiated a new era of investigation, with the purpose of disclosing the conundrum functions behind EVs in fungi.

Currently, EVs have been isolated from many different cell types and fluids [9], as well as in a wide range of fungal species, including yeast species such as *Histoplasma capsulatum*, *Sporothrix schenckii*, *Candida parapsilosis*, *Saccharomyces cerevisiae*, *Malassezia sympodialis*, *Paracoccidioides brasiliensis*, *Candida albicans*, *Pichia fermentans*, *Cryptococcus gattii*, *Sporothrix brasiliensis*, *Paracoccidioides lutzii*, *Candida glabrata*, *Candida tropicalis*, *Talaromyces marneffei*, and *Candida auris* [10,11,12,13,14,15,16,17,18,19,20,21,22], along with a set of filamentous species, including *Exophiala dermatitidis*, *Alternaria infectoria*, *Trichophyton interdigitale*, *Rhizopus delemar*, *Fusarium oxysporum f.* sp. *Vasinfectum*, *Trichoderma reesei*, *Aspergillus fumigatus*, *Aspergillus flavus*, and *Penicillium digitatum* [23,24,25,26,27,28,29,30].

The discovery that RNAs are contained in EVs, mainly related to microRNA (miRNA) specimens in the years 2006–2007, paved the way for investigating the role of EVs as mediators of cell-to-cell communication [31,32]. This finding represents a new paradigm of the production and release of EVs, which have been considered for many years as “platelet dust”—i.e., cellular debris of damaged cells [33]—or an export system activated to maintain cellular homeostasis [34].

Recently, the participation of EVs in environmental signaling has been proposed [35]. Milieu sensing in fungi is a finely tuned mechanism that ensures fungal survival and the establishment of infection [36]. Based on EV content, these molecules support fungal pathophysiology with the regulation of intracellular matrix component biosynthesis [37], capsule formation [38], and cell wall construction [39]. Furthermore, quorum-sensing molecules within EVs may be involved in regulating the morphology of *C. albicans* [40]. Another level of regulation mediated by EVs is ensured by their RNA content. It has been demonstrated that EVs are loaded with different classes of functional RNAs such as miRNA, messenger RNA (mRNA), ribosomal RNA (rRNA), transfer RNA (tRNA), small RNA (sRNA), and long non-coding RNA (lncRNA) [41].

Many aspects of the pathophysiology of fungal infections might be influenced by the transport of RNA molecules within EVs. The uptake of fungal RNA-EVs by host cells or even fungal cells is conceivably involved in gene regulation and, consequently, triggers changes in the responses of the recipient cells. From this perspective, the involvement of EVs in bidirectional communication has been proposed [42,43] and, currently, in intraspecies cellular communication, in which the incorporation of EVs by fungal cells has been shown to be critical for governing aspects of virulence and resistance toward fungal agents [17,39,44]. However, a deeper understanding of the molecular mechanisms underlying this regulation requires further investigation.

In this review, we describe the current knowledge on RNA content within EVs, along with the influence of these molecules on two-way systems, such as the fungus–host interactions and intraspecies regulation. We also discuss the challenges in RNA-EV work and raise hypotheses of the cellular strategies underlying this RNA export system.

## 2. RNA Content of Fungal EVs

The presence of RNAs within extracellular vesicles was first described in 2007 [32]. The authors demonstrated that RNAs obtained from mammalian EVs were delivered to another cell in a functionally activated manner—evidence of a new mechanism of intercellular communication mediated by EVs that relies on RNA transference [32,45]. This RNA-EV trafficking is termed exosomal shuttle RNA (esRNA), and comprises mRNAs and an enrichment of miRNA content [32]. The predictive combinatory interactions of the 121 miRNAs identified in this study displayed a putative potential to regulate approximately 24,000 mRNAs, revealing a widespread coordinated regulation. Moreover, the characterization of RNAs loaded in EVs found an absence or minimal presence of ribosomal RNAs and an abundance of small RNAs, including the 19–22 nt class of non-coding RNAs.

The first study addressing fungal EV RNA content was reported in 2015 [46]. In this study, a comparative analysis of RNA specimens from human pathogenic fungi—such as *C. albicans*, *C. neoformans*, and *P. brasiliensis* (isolate Pb18)—as well as the yeast model *S. cerevisiae* was conducted.

This solid comparative analysis of RNA-containing vesicles in pathogenic and non-pathogenic fungal species revealed the presence of RNAs with heterogeneous sizes, mostly shorter than 250 nt, primarily corresponding to small non-coding RNA sequences and miRNA-like sequences, while also demonstrating the co-purification of a small percentage of mRNAs (approximately 10%) [46]. Accordingly, the data displayed a high abundance of miRNAs, with a total of 1242 miRNAs being identified and classified as follows: 145 for *P. brasiliensis*, 344 for *C. neoformans*, 423 for *C. albicans*, and 532 for *S. cerevisiae.* In addition, 47 miRNAs exhibited differential distribution, with higher levels of miRNA transcripts in *C. neoformans*, followed by *P. brasiliensis*, *C. albicans*, and *S. cerevisiae.* Only 20 miRNAs were shared among all studied species. Unique miRNA sequences were detected in *P. brasiliensis*, including has-mir5685, cre-MIR905, dre-MIR-125-a2, and has-mir-5583-1; and in *S. cerevisiae*, with ame-mir-3797 and cin-mir-4104. Of the 114 identified sRNA sequences, 11 were common among all studied species. Additionally, eight sequences corresponding to the lncRNAs were identified. Among these, ICR1 and PWR1 are involved in upstream signaling circuits that regulate yeast transition, whereas short non-coding RNAs (sncRNAs) play a role in mRNA and rRNA modifications that ultimately affect cell growth. Moreover, four sncRNAs have been described (LSR1, snR19, snR16, and snR14) as components of the spliceosome core, with functions in pre-mRNA processing. Indeed, the spliceosome machinery and pre-mRNA processing are critically involved in fungal adaptation to different niches, as well as their pathogenic capability [47,48].

This comparative analysis opens new paths for investigating fungal pathophysiology by tracking differences in EV-associated RNA molecules derived from species of fungi with different abilities in terms of human association and pathogenicity. These analyses allow the determination of peculiarities in composition and abundance, which may reflect the levels of regulation for niche preferences, adaptation, and virulence. Since then, EV RNAs from other fungal species—including *H. capsulatum* [49], *C. auris* [22], and *M. sympodialis* [50]—have been identified.

In this context, another comparative study was performed to assess the RNA content of EVs from different isolates of *P. brasiliensis* (Pb18 and Pb3) and *P. lutzii* (Pb01 isolate) [18]. Both are agents of human paracoccidioidomycosis (PMC), and are endemic species in Latin America—mainly in Brazil. Isolates P18 and Pb3 are phylogenetically distributed in groups S1 and PS2, respectively. These species have different profiles of virulence and immune response. The data obtained in this study revealed the presence of the following classes of RNAs: mRNA, lncRNA, sncRNA, and rRNA. A high number of sncRNA molecules was observed, with 25 nt extensions, and certain molecules displayed the interesting ability to align to a particular exonic mRNA region (5′, 3′, or in the middle); hence, these molecules were named “exonic sRNA”. In this analysis, the greatest abundance of exonic sRNAs was found in Pb18 (104), followed by Pl01 (27) and Pb3 (19). Moreover, unique exonic sRNAs were identified in Pb18 (89), Pb01 (21), and Pb3 (4). The assumption is that exonic sRNAs in EVs provide sRNA interference (ex-siRNA) and regulate the RNA interference (RNAi) machinery [18]. Herein, we speculate that exonic sRNAs could potentially be a set of mature miRNAs loaded within the EVs. The reason behind our speculation is that either of these molecules might interact with the 3′ UTR, 5′ UTR, and/or coding sequence. The interaction with different binding regions can lead to a repertoire of gene-regulatory activities, such as repression of translation, activation of translation, or transcription regulation [51].

**Figure 1:**
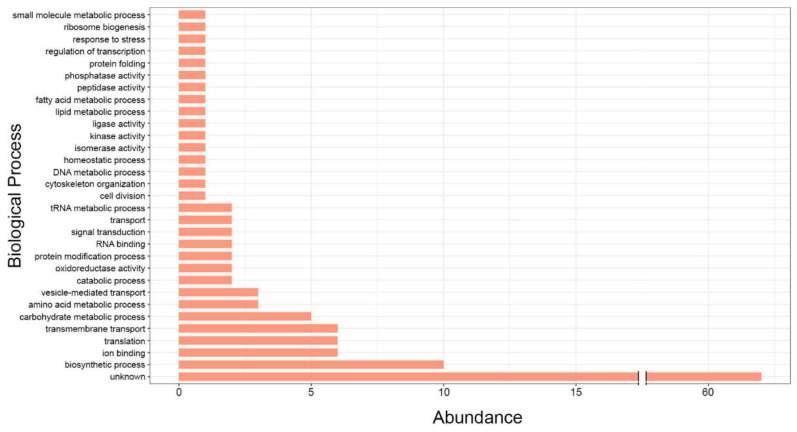
Functional categorization of exonic sRNAs identified in *P. brasiliensis* according to Gene Ontology (GO) terms within the biological process subclassification.

The listed exonic-sRNA-targeted genes described in *P. brasiliensis* [18] include α-amylase (PADG_04422), which is potentially involved in the synthesis of α-glucan—a key element in the fungal cell wall—and β-glucanase (PADG_04922), which cleaves β-glucan in the cell wall—a ligand of dectin-1—and is involved in immune recognition. Furthermore, a comparative Pb study assessed the regulation of dendritic cells by indirect contact with *P. brasiliensis* (Pb18) and, potentially, its EVs in an elegant model. The data showed downregulation of transcription factors such as Gabpb2 and Pknox1, which are involved in lymphopoiesis [52] and hematopoietic stem and progenitor cell activity [53]. From these results, Pb EVs were assumed to modulate the immune response in a favorable manner in the early stages of fungal infection. Herein, to demonstrate the striking regulation promoted by these exonic sRNAs, we compiled a set of discovered exonic sRNAs and clustered them with their putative target genes. Then, we classified these regulated genes within the associated biological processes in accordance with Gene Ontology (GO) terms, showing that Pb exonic sRNAs contained in EVs potentially regulate the following processes in fungal cells: carbohydrate, lipid, fatty acid, and amino acid metabolism; vesicle trafficking; signal transduction; protein folding; and nucleic and biosynthetic processes (Supplementary Table S1 and Figure 1). From this projection, the autoregulatory function of fungal EVs in the cellular metabolism and cell communication, which reinforces the role of RNA-EVs in interkingdom and intraspecies signaling, is evident. Indeed, a recent study demonstrated the regulatory function of fungal EVs in fungal gene expression, governing the pathophysiological attributes of the pathogenic fungi *C. albicans, A. fumigatus*, and *P. brasiliensis* [54]. However, whether exonic sRNAs could be mature miRNAs exported by fungal EVs to confer faster adaptive responses requires further study and elucidation.

Furthermore, another study reported the EV RNA content of two isolates of *H. capsulatum* (G186AR and G217B) [49]. *H. capsulatum* is the causative agent of histoplasmosis, and represents a global concern, affecting both immunocompetent and immunocompromised patients. The strains G186AR and G217B show differences in virulence properties and cell wall composition. In this study, the authors revealed the occurrence of mRNAs and ncRNAs, with differences in their abundance between the studied isolates. Among ncRNAs, a large set of tRNAs was identified. Moreover, miRNA-like RNAs with regulatory functions were predicted by analyzing the putative development of secondary structures within the EV RNA content. Another query addressed by Alves et al. (2019) [49] was the correlation between yeast-associated and EV-associated RNAs, and the data showed no or weak correlation between the contents from different sources, strengthening the hypothesis that a fine-tuned mechanism directs RNAs in EVs and their regulatory functions.

In another line of investigation, Rayner et al. (2017) [50] assessed the small RNA content in *M. sympodialis* under different pH ranges, in an attempt to mimic the pH changes during atopic eczema (AE)—a disease commonly triggered by this fungus. Although the approach was very interesting, no significant difference in RNA-associated EVs was shown for the assessed conditions, although more than 300 non-coding features were mapped for this species [50].

Recently, a study profiled the cellular and EV transcriptomes of *C. auris* [22]—a fungus classified as a global public health threat. This species requires special care because it is characterized by remarkable antifungal resistance against different chemical classes of compounds, such as polyenes, azoles, and echinocandins. Two strains of *C. auris* (B8441 and MMC1) were used, and their RNA content was evaluated after fungal exposure to caspofungin. The authors demonstrated that EV concentration was twofold higher during caspofungin treatment than that in untreated conditions. Although mRNA molecules were identified, the bulk comprised sRNAs (<200 nt in length), including ncRNAs, tRNAs, and fragments of mRNA. The authors also demonstrated a different profile of EV transcripts in caspofungin treatment versus untreated conditions. Moreover, a 10x higher yield of RNA was observed for caspofungin treatment.

**Figure 2:**
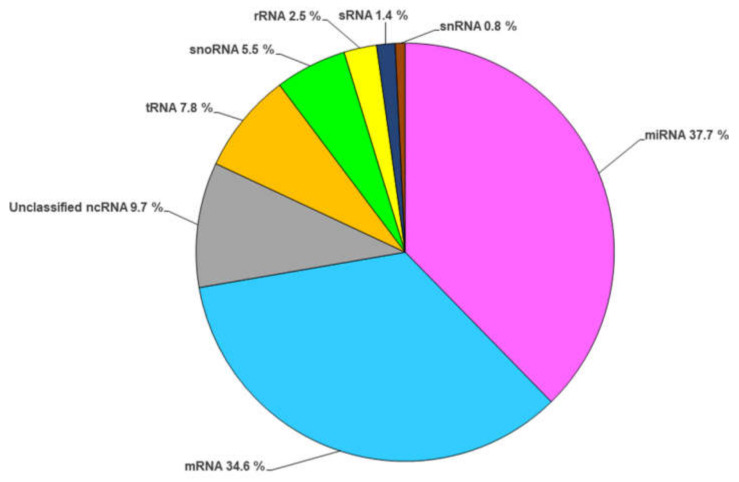
A compilation of data on the abundance of RNAs (%) loaded in extracellular vesicles in a set of fungal species, including *C. albicans*, *C. neoformans*, *S. cerevisiae*, *C. auris*, *H. capsulatum*, *M. sympodialis*, *P. brasiliensis*, and *P. lutzii*.

Herein, we intended to summarize the current information about the RNA contents in EVs, and with this purpose we carried out a deep survey of the published data about RNAs loaded in fungal EVs, and our data shed light on the prevalence of regulatory RNAs (about 65.5%) in comparison with mRNAs (about 34.5%), as shown in Figure 2. The distribution of these regulatory molecules was as follows: miRNAs (about 1480 molecules), ncRNAs (about 400 molecules), tRNAs (about 300 molecules), small nucleolar RNA (about 220 molecules), sRNA (about 56 molecules), and small nuclear RNA (about 30 molecules). These data were based on the RNA characterization of the following fungal species: *C. albicans*, *C. neoformans*, *S. cerevisiae* [46], *C. auris* [22,55], *H. capsulatum* [49], *M. sympodialis* [50], *P. brasiliensis*, and *P. lutzii* [18]. Moreover, we also outlined the distribution of the regulatory RNA molecules within each analyzed species (Supplementary Figure S1). miRNA was the main regulatory molecule loaded in fungal EVs, except for *C. auris,* wherein tRNA was the most abundant molecule. A proper understanding of the RNA content in EVs, the changes in the signaling pathways that regulate these transcripts, and the targeted genes may reveal strategies employed during the interaction between systems, and also shed light on alternatives to tackle fungal arming during the infection.

## 3. The Signaling Role of Fungal RNA-EVs during Host–Pathogen Interaction

The role of EVs in cross-kingdom communication is unambiguously accepted, and it is conceivable that this mechanism is mainly achieved because EVs work as vehicles for hijacking sRNAs in a two-way manner; therefore, EVs are also termed “Trojan horses” [56]. The function of sRNAs in the regulation of gene expression is based on the RNAi principle, which comprises the interplay of a commander RNAi and an Argonaute (AGO) protein that selectively silences targeted genes. Notably, EVs from *H. capsulatum* contain RNA-binding proteins, one of which belongs to the RNAi machinery [49]. Thus, there are two possible ways in which these sRNAs regulate targeted genes: either by association with their own sources of RNAi machinery, or by association with AGO proteins in recipient cells.

Previously, it was demonstrated that the secretion of exosome-containing miRNA and AGO protein by the gastrointestinal parasite *Heligmosomoides polygyrus* neutralizes host responses and favors infection [57]. However, many questions have arisen since then, mainly concerning how this transfer between cell-walled organisms could be possible. This motivated an elegant study carried out by Cai et al. (2018) [58] using a co-culture of *Arabidopsis* and *Botrytis cinerea*. In this study, the authors tracked the accumulation of plant EVs in fungal cells, profiled the sRNAs produced during infection, and showed that the target silenced genes were related to fungal virulence traits. Moreover, other studies have shown the enhancement of sRNAs in cotton plants after fungal infection, reinforcing their role in regulating the expression of opponents and warding off infection [59]. Similarly, sunflower EVs incubated with *S. sclerotiorum* spores were taken up by fungal cells and, as a result, these EVs had a significant impact on fungal morphology and hyphal development. EV-treated cells presented a strong reduction in hyphal growth, abnormalities in hyphal shape with a decrease in length followed by curly/wave hyphae, and the presence of non-germinated spores [60]. Conversely, fungal RNA-EVs can also suppress host defense. In pathosystem infection models, such as *Botrytis cinerea* co-cultured with *Arabidopsis* and tomato, the hijacking of Bc-sRNAs promoted the suppression of host genes, including mitogen-activated protein kinase (MPK), oxidative stress responsive gene, peroxiredoxin (PRXIIF), and cell-wall-related kinase (WAK), leading to an enhancement in plant susceptibility, ascribed to a tight involvement of host immunity against fungal infection [61]. Taking advantage of this information, another study carried out by Wang et al. (2016) [62] showed that the double mutation of Bc dicer1/2 genes in *Botrytis cinerea* attenuates its virulence in different recipient cells, including fruits, vegetables, and flower petals. Moreover, the authors developed a Bc-DCL1/2–RNAi *Arabidopsis* plant system, and the RNAi plants displayed lower susceptibility to *B. cinerea*. These results provide insights into the development of strategies to control plant diseases using RNAi effectors and delivery systems.

In addition, the entomopathogenic fungus *Beauveria bassiana* secretes a miRNA-like RNA (bba-miR1) loaded into its vesicles, targeting the *Anopheles stephensi* mosquito to suppress the expression of Toll receptor ligand Spätzle 4 (Spz4), attenuates the immune response, and favors fungal infection [63]. Once again, understanding the mechanisms underlying miRNA delivery is crucial to control threats to human health and crops, and could be exploited as an alternative to develop new therapies.

## 4. Advancements in Fungal RNA-EV Research

**Figure 3:**
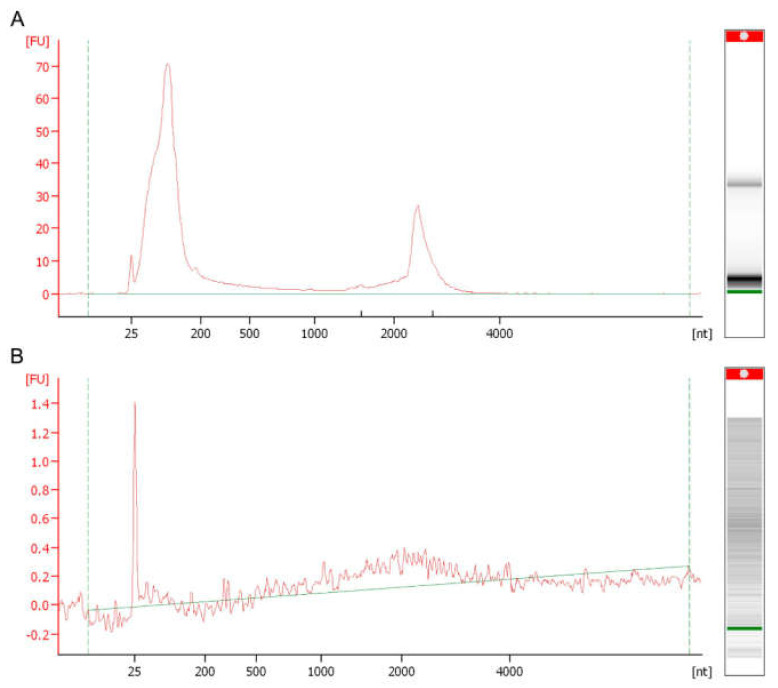
Scheme of RNA-EV visualization by electrophoretic profiling: (**A**) RNAs loaded in EVs from *C. haemulonii*. (**B**) RNAse-treated samples to illustrate EV RNA degradation.

Undoubtedly, the investigations of sRNAs within EVs break paradigms and challenge researchers in many respects regarding RNA quality inspection and the strategies employed to validate that these molecules are not loosely aggregated with EV membranes, but are loaded within these vesicles. In this respect, controls employing EVs undergoing RNAse treatment before sRNA extraction, or the rupture of EVs with Triton X-100 followed by nuclease digestion, were used to track some features of these molecules, and to prove that they were packaged within EVs [32,57]. Notably, the migration analysis of these molecules in microfluidic electrophoresis displays another striking feature that might lead to a misconception of RNA degradation. Usually, the profile obtained in electropherograms depicts peaks that appear mainly in the range of 25–200 nt, which are correlated with fragments of sRNA content, as schematically represented in Figure 3. Moreover, a common issue in the fungal RNA-EV work process is the low yield during extraction procedures, which impairs the downstream steps of analysis and library construction. To address these issues, an alternative is to create a pool of concentrated EVs before RNA extraction. Choosing a suitable library is critical when investigating RNA-EVs; in this regard, it is noteworthy to consider the proportions of each RNA type while not restricting the library to mRNAs. However, constructing a library and working on different fragment sizes would be a promising approach. Further methodological publications addressing these points and offering new possibilities are required to counteract the issues associated with EV RNAs in fungi.

## 5. Conclusions and Future Perspectives

**Figure 4:**
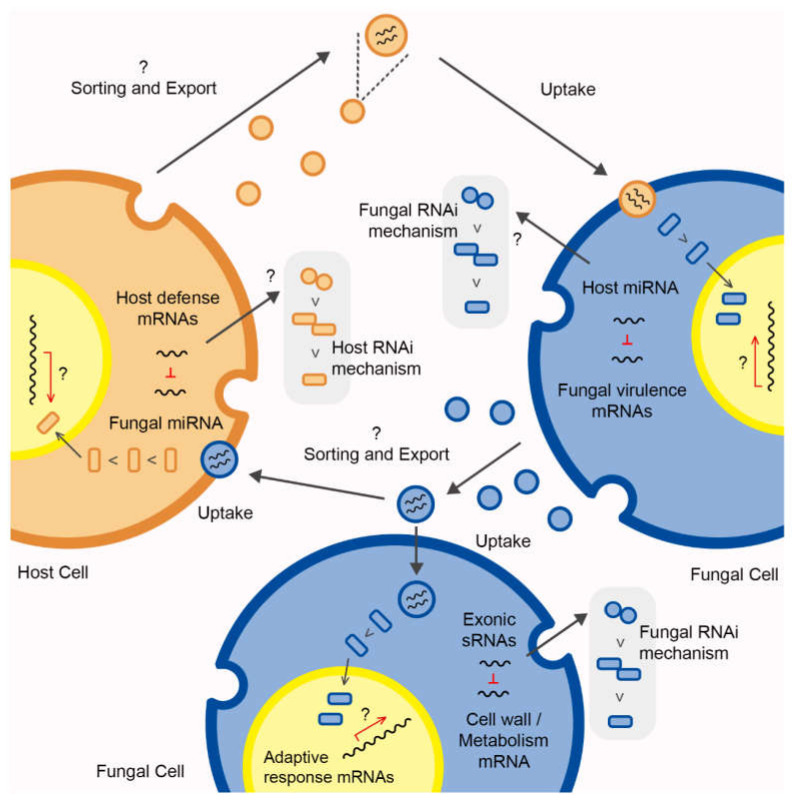
Extracellular vesicles’ (EVs) RNA transference between host and fungal cells via bidirectional communication events and intraspecies RNA transference mediated by fungal EVs. The sRNAs/miRNAs’ transference leads to target mRNA silence and, concomitantly, suggests paths able to regulate the gene expression, and affects cellular behavior in recipient cells. The question marks represent gaps that require further investigation, such as the signaling mechanisms by cells to sort molecules within EVs in response to extracellular stimuli, paths that might coordinate gene modulation after EVs uptake, and the conceivable use of RNAi machinery from recipient cells to trigger gene silencing and other regulatory functions.

Although RNA-containing EVs are protagonists during signaling and communication between cells, the precise regulation promoted by these molecules is not clearly understood. It is possible to speculate on some points when examining the content of these molecules loaded in EVs. First, packing and transferring rRNA is not the goal, since the recipient cells might be responsible for the translation task in a more efficient and energetically favorable manner. Second, although the presence of functional mRNAs was identified in EVs from *P. brasiliensis* and *P. lutzii*, a higher abundance was observed for regulatory RNAs, such as sRNAs, miRNAs, and tRNAs, and it is highly conceivable that these molecules interact with the owner and/or host AGO proteins coordinating the host RNAi machinery. Understanding these tightly regulated processes that underlie EV content and trafficking opens new paths for investigation. Tracking of EV RNA can shed light on the plasticity, virulence, and niche preferences of some fungal species or strains. By exploiting this knowledge, researchers might be able to “edit” the framework mechanism in RNA production and transference that ultimately alters the circuit of information to disarm and ward off pathogens. A great improvement in systems such as host-induced gene silencing (HIGS) technology might be achieved for safer use in the biological pest control of crops, as well as an enhancement in therapeutic strategies to control infectious agents that threaten human health. However, the mechanistic framework for the regulation of fungal EV-containing sRNAs requires further investigation. Several questions arise from the current data: What determines RNA sorting in EVs? Which signaling pathways may cross to activate EV production and sorting? Which molecular mechanism underlies the EV RNA regulation of target genes in recipient cells? A schematic overview of EV RNA transference knowledge and the queries that await for further investigation is shown in Figure 4.

## Data Availability

Not applicable.

## Supplementary Materials

The following supporting information can be downloaded at: https://www.mdpi.com/article/10.3390/cells11142184/s1, Table S1: An overview of RNA-containing fungal EVs; Figure S1: Distribution of regulatory EV RNA molecules between the following fungal species: *C. albicans*, *C. neoformans*, *S. cerevisiae*, *C. auris*, *H. capsulatum*, *M. sympodialis*, and *Paracoccidioides* spp.
