# Supplementary figures and images for: The RNA Content of Fungal Extracellular Vesicles: At the “Cutting-Edge” of Pathophysiology Regulation
Source: Cells. 2022 Jul 13;11(14):2184. doi: 10.3390/cells11142184 (PMC9318717; doi:10.3390/cells11142184)

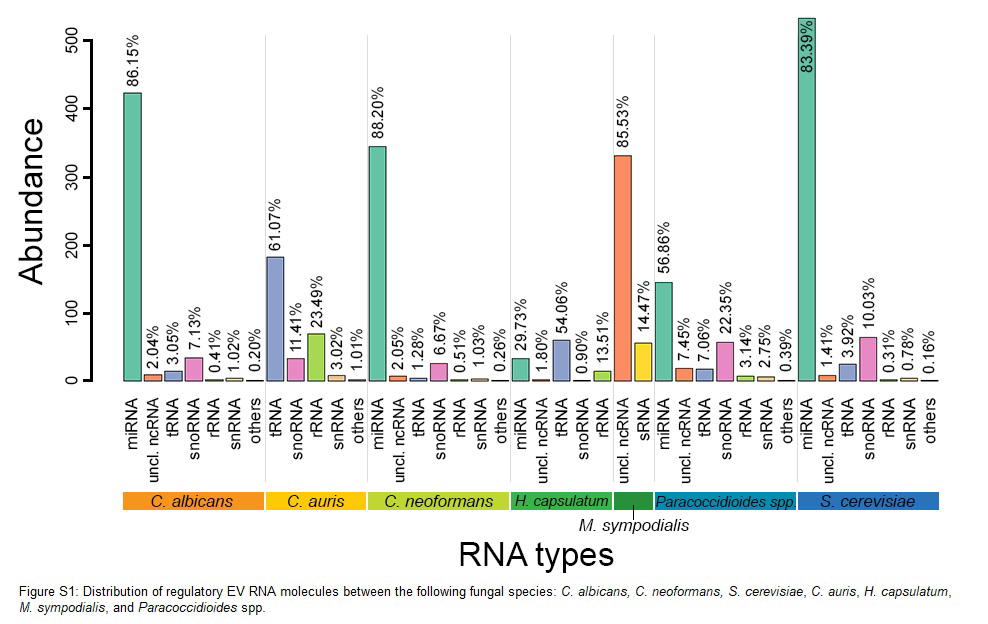

Supplement: Supplementary file 1 [file cells-11-02184-s001.zip › Supplementar_Fig_S1.Distribution of regulatory EV RNA molecules.tif]
